# Enhanced fear memories and brain glucose metabolism (^18^F-FDG-PET) following sub-anesthetic intravenous ketamine infusion in Sprague-Dawley rats

**DOI:** 10.1038/s41398-018-0310-8

**Published:** 2018-11-30

**Authors:** Kennett D. Radford, Thomas Y. Park, Shalini Jaiswal, Hongna Pan, Andrew Knutsen, Michael Zhang, Mercedes Driscoll, Lisa A. Osborne-Smith, Bernard J. Dardzinski, Kwang H. Choi

**Affiliations:** 1 Daniel K. Inouye Graduate School of Nursing, Uniformed Services University of the Health Sciences, Bethesda, MD 20814 USA; 2 Department of Psychiatry, Uniformed Services University of the Health Sciences, Bethesda, MD 20814 USA; 3 Center for the Study of Traumatic Stress, Uniformed Services University of the Health Sciences, Bethesda, MD 20814 USA; 4 Center for Neuroscience and Regenerative Medicine, Uniformed Services University of the Health Sciences, Bethesda, MD 20814 USA; 5 National Capital Consortium Psychiatry Residency Program, Walter Reed National Military Medical Center, Bethesda, MD 20814 USA; 6 Nurse Anesthesia Program, Oregon Health and Science University, Portland, OR 97239 USA; 7 Department of Radiology and Radiological Sciences, Uniformed Services University of the Health Sciences, Bethesda, MD 20814 USA

## Abstract

Ketamine is a multimodal dissociative anesthetic, which provides powerful analgesia for victims with traumatic injury. However, the impact of ketamine administration in the peri-trauma period on the development of post-traumatic stress disorder (PTSD) remains controversial. Moreover, there is a major gap between preclinical and clinical studies because they utilize different doses and routes of ketamine administration. Here, we investigated the effects of sub-anesthetic doses of intravenous (IV) ketamine infusion on fear memory and brain glucose metabolism (BGluM) in rats. Male Sprague-Dawley rats received an IV ketamine infusion (0, 2, 10, and 20 mg/kg, 2 h) or an intraperitoneal (IP) injection (0 and 10 mg/kg) following an auditory fear conditioning (3 pairings of tone and foot shock [0.6 mA, 1 s]) on day 0. Fear memory retrieval, fear extinction, and fear recall were tested on days 2, 3, and 4, respectively. The effects of IV ketamine infusion (0 and 10 mg/kg) on BGluM were measured using ^18^F-fluoro-deoxyglucose positron emission tomography (FDG-PET) and computed tomography (CT). The IV ketamine infusion dose-dependently enhanced fear memory retrieval, delayed fear extinction, and increased fear recall in rats. The IV ketamine (10 mg/kg) increased BGluM in the hippocampus, amygdala, and hypothalamus, while decreasing it in the cerebellum. On the contrary, a single ketamine injection (10 mg/kg, IP) after fear conditioning facilitated fear memory extinction in rats. The current findings suggest that ketamine may produce differential effects on fear memory depending on the route and duration of ketamine administration.

## Introduction

First responders and medics administer ketamine, a multimodal dissociative anesthetic, to the traumatically injured to provide sedation and analgesia without compromising hemodynamic stability and spontaneous respirations^1^. Clinicians also administer ketamine via a continuous intravenous (IV) infusion at sub-anesthetic doses to alleviate acute pain and reduce opioid consumption in the post-operative period^2^. Despite apparent advantages as a trauma anesthetic, ketamine can produce psychomimetic effects that raise concerns for potential adverse effects on long-term psychological health^3,4^. Trauma survivors diagnosed with post-traumatic stress disorder (PTSD) often suffer from intrusive fear memories that fail to extinguish^5–7^ and a dysfunctional glutamatergic signaling pathway has been implicated in these symptoms^8^. Because ketamine is known to dysregulate glutamate signaling through *N*-methyl-d-aspartate (NMDA) receptor antagonism^9^, it is important to understand the impact of ketamine administration in the peri-trauma period on traumatic memory processing and expression.

Clinical investigations exploring the relationship between peri-trauma ketamine administration and PTSD have shown conflicting results^10–12^. A retrospective review of U.S. military service members who suffered severe combat-related burns and received intraoperative ketamine administration found no association between ketamine administration and PTSD diagnosis^10^. However, that study did not report elapsed time from the injury to ketamine administration, co-administered psychoactive medications, or comorbid injuries associated with PTSD such as mild traumatic brain injury. In contrast, patients who received ketamine analgesia immediately after traumatic injury reported increased dissociation, acute stress disorder (ASD), and PTSD symptoms compared with those who received non-ketamine analgesics^13^. Also, adverse effects of ketamine were reported describing increased rates of PTSD among burn patients who received ketamine analgesia as compared with those who did not receive ketamine treatment after the injury^14^.

Results from preclinical studies investigating the effects of ketamine on fear memory in rodents are also inconsistent. Previous studies, utilizing intraperitoneal (IP) ketamine injection following fear conditioning, reported either improved^15,16^, worsened^17–20^, or no effects^21,22^ on fear memory of rodents. For instance, mice that received a ketamine injection (30 mg/kg, IP) at 1 h or 1 week after fear conditioning showed no change^23^, while ketamine injected 1 day after fear conditioning facilitated fear memory extinction in rats^16^. Conflicting results from these studies may be due to different ketamine doses, timing of injection after fear conditioning, and experimental procedures utilized in the studies.

There is a major gap between clinical and preclinical studies on ketamine. Although IV ketamine is predominantly used in patients, most preclinical studies inject IP ketamine to animals, which reduces the translational value of these studies. A bolus IP injection of ketamine does not maintain a steady-state plasma drug concentration over time. Following an IP injection in rodents, ketamine plasma levels rapidly peak and decline secondary to drug distribution and elimination^24–26^. In comparison, a continuous IV ketamine infusion maintains consistent elevated plasma drug levels over an extended period of time^27^, which allows for sustained impact on neurobiological mechanisms in the brain. To our knowledge, there are no previous reports of the effects of IV ketamine infusion on fear memory retrieval, extinction, and recall in rodents.

Identifying specific brain regions that are sensitive to IV ketamine infusion and fear memory retrieval can assist researchers and clinicians to interpret behavioral effects of ketamine. Previous studies using ^18^F-fluoro-deoxyglucose positron emission tomography (FDG-PET) found that an IV ketamine infusion altered regional brain glucose metabolism (BGluM) in depressed patients. For instance, a ketamine infusion (0.5 mg/kg, IV) decreased BGluM in the habenula and amygdala of depressed patients^28^. Two hours after the ketamine infusion (0.5 mg/kg, IV), the BGluM in the dorsal anterior cingulate cortex and hippocampus was increased, whereas it was decreased in the orbitofrontal cortex of depressed patients^29^. In healthy volunteers, a ketamine infusion (0.77 mg/kg) induced psychotic symptoms and increased BGluM in both the prefrontal cortex (PFC)^30^ and anterior cingulate cortex^31^. A preclinical study with ex vivo 2-deoxyglucose (2-DG) autoradiography reported that IV ketamine (10 mg/kg, 2–5 min) decreased BGluM in the sensory motor cortex while increasing it in the cingulate gyrus, hippocampus, globus pallidus, and substantia nigra of rats^32^. However, the effects of a longer IV ketamine infusion on in vivo BGluM of animals are not reported.

The main goal of this study was to investigate the effects of sub-anesthetic doses of IV ketamine infusion on fear memory and brain glucose utilization. Another goal was to compare the effects of IV infusion and IP injection of ketamine on fear memory in rats.

## Materials and methods

### Animals

Adult male Sprague-Dawley rats (Envigo Laboratories, Dublin, VA) weighing 300–315 g were used. A jugular venous catheter (3Fr, polyurethane; Instech, Plymouth Meeting, PA) was surgically implanted under isoflurane anesthesia at the Envigo Laboratories (Dublin, VA) prior to animal arrival. The catheter was tunneled under the skin and connected to a vascular access button (Instech, Plymouth Meeting, PA) that exited the dorsal position between the front rodent scapulae. Rats were single housed in clear Plexiglas shoebox cages in a climate-controlled environment with food/water ad libitum. Animals were habituated to a 12-h reversed dark/light cycle (light off at 0600 hours; testing during the dark cycle) and handled daily for 7 days prior to testing. Catheters were flushed once every 3 days to verify venous patency and locked with 0.1 mL heparin/glycerol solution (Braintree Scientific, Braintree, MA). All procedures were performed in accordance with the National Institutes of Health Guide for the Care and Use of Laboratory Animals and approved by the Institutional Animal Care and Use Committee at the Uniformed Services University of the Health Sciences (Bethesda, MD).

### Auditory fear conditioning

**Fig. 1:**
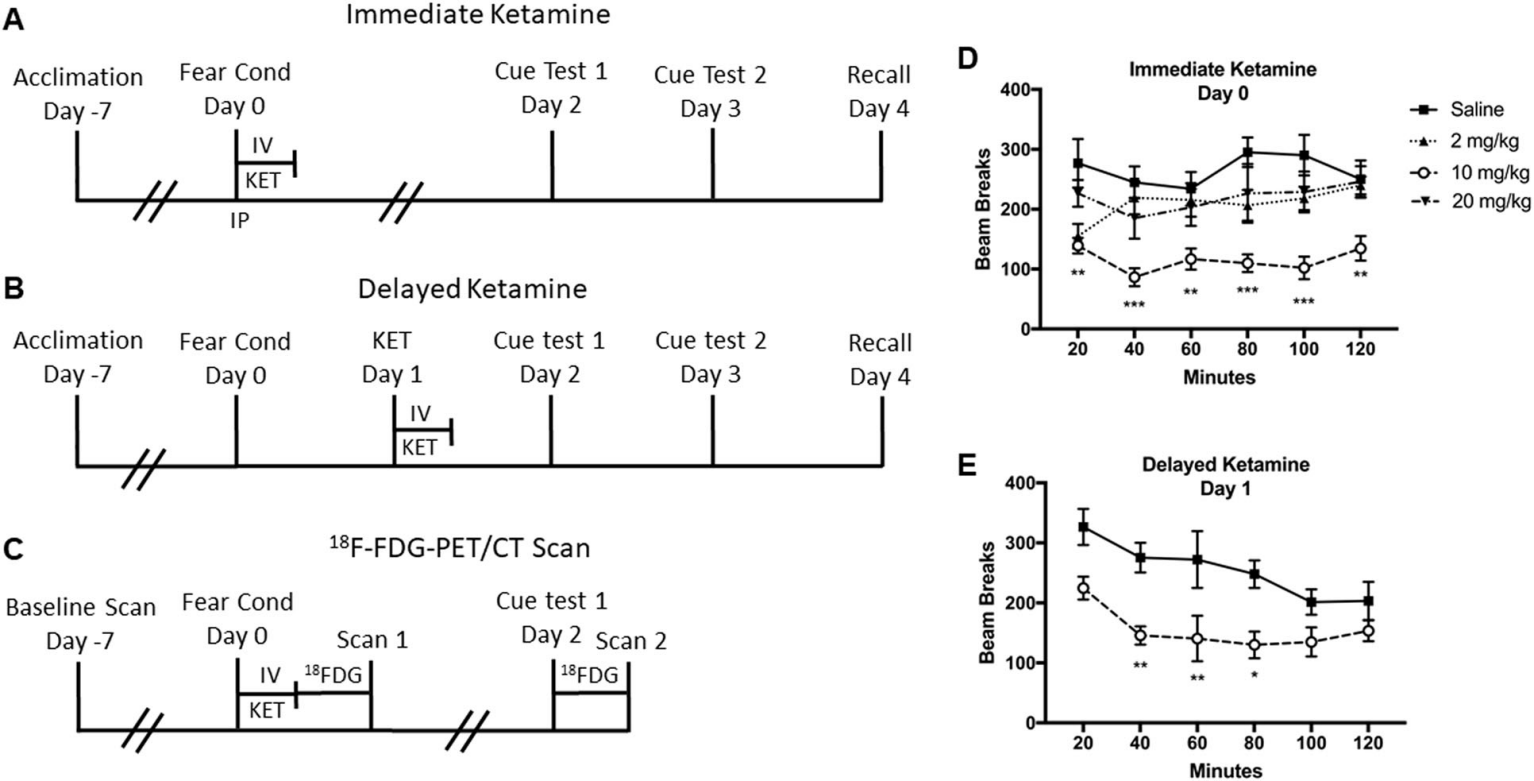
Experimental design and locomotor activity during the IV ketamine infusion. **A** Immediate ketamine experiment. Animals were subjected to fear conditioning in Context A followed by a 2-h IV ketamine infusion (0, 2, 10, or 20 mg/kg) or single IP ketamine injection (0 or 10 mg/kg) on day 0. On days 2 and 3, animals were tested on cued fear retrieval and extinction, which consisted of 12 auditory tone presentations without the foot shock in Context B. Fear memory recall was tested in Context A on day 4. **B** Delayed ketamine experiment. Animals were subjected to fear conditioning on day 0 and received a 2-h IV ketamine infusion (0 or 10 mg/kg) on day 1. Animals were tested on cued fear retrieval and extinction on days 2 and 3, followed by fear recall testing on day 4. **C** FDG-PET and CT experiment. Animals underwent a baseline FDG-PET/CT scan, 1 week before the fear conditioning and ketamine infusion. Animals were subjected to fear conditioning followed by a 2-h IV ketamine infusion (0 or 10 mg/kg) on day 0. Immediately after the ketamine infusion, animals were scanned with ^18^F-FDG-PET/ CT (Scan 1). On day 2, animals were tested on cued fear memory retrieval and extinction, and then scanned with the ^18^F-FDG-PET/CT (Scan 2). **D** Spontaneous locomotor activity during the ketamine infusion given immediately after fear conditioning. The ketamine infusion (10 mg/kg) significantly reduced locomotor activity during the infusion period. **E** The ketamine infusion (10 mg/kg) given 1-day after fear conditioning also reduced locomotor activity during the infusion period. Data are shown as mean ± SEM (*p *<* 0.05, **p *<* 0.01, ***p *<* 0.001 vs. saline controls)

The fear conditioning experiment was carried out in a chamber (Context A) constructed with Plexiglas and aluminum walls (Coulbourn Instruments, Lehigh Valley, PA) on day 0 (Fig. 1). The chamber contained a metal grid floor connected to a shock generator (Coulbourn Instruments, Lehigh Valley, PA) with a wall-mounted speaker to provide the auditory stimuli and a dim light (2–3 lux). A computer interface program (Coulbourn Instruments, Lehigh Valley, PA) controlled the delivery of foot shock and auditory stimuli. An infrared video camera mounted above the chamber facilitated video recording and observation. The chamber was housed inside a larger sound-attenuating box (Coulbourn Instruments, Lehigh Valley, PA) with a background noise level of 55 dB.

Rats underwent a fear conditioning procedure as previously described^33^. After a 180-s acclimation period, rats received three pairings of an auditory tone (5 kHz, 75 dB, 20 s) that co-terminated with a mild foot shock (0.6 mA, 1 s). A variable inter-trial interval (ITI) that ranged from 90 to 120 s was used to prevent tone prediction by animals. Animals were removed from the chamber 60 s after the third tone-foot shock pairing and received either an immediate IV infusion, immediate IP injection, or a delayed IV ketamine infusion 1 day after the conditioning (Fig. 1). The conditioning chamber was cleaned with 70% ethanol and thoroughly dried after each use.

### Ketamine infusion

Racemic (±) ketamine hydrochloride (100 mg/mL) (Mylan Institutional LLC, Rockford, IL) was diluted in 0.9% sterile saline and was administered IV in operant conditioning chambers (Med Associates Inc., St. Albans, VT). Each chamber (14ʺ L × 12ʺ W × 15ʺ H) was equipped with an infusion pump (Harvard Pump 11 Elite, Holliston, MA) using a 5 mL Hamilton glass syringe connected to a fluid swivel (Instech, Plymouth Meeting, PA) by polyurethane tubing encased in a metal spring-wire tether to prevent chewing by the animals. The spring-wire tether was attached to the vascular access button on the rat using a luer-lock connection. All tethered animals had free mobility in the chambers during the infusion period. Each infusion chamber was equipped with two infrared photo-beams that monitored spontaneous horizontal activity of animals during the infusion period. The number of beam breaks was recorded by the computer during the infusion period.

### Immediate ketamine experiment

Animals were randomly assigned to four groups, which consisted of ketamine doses 0 (*n* = 11), 2 (*n* = 9), 10 (*n* = 11), and 20 mg/kg (*n* = 11) infused over 2 h. Immediately after fear conditioning on day 0, ketamine groups received a ketamine IV bolus to facilitate ketamine plasma loading prior to the initiation of the 2-h infusion (Fig. 1A). Bolus ketamine doses were 0.5 mg/kg for the 2 mg/kg group and 2 mg/kg for the 10 and 20 mg/kg groups. The sub-anesthetic IV ketamine bolus and infusion doses were determined based on our previous study^27^. The saline control group received a saline bolus followed by a 2-h saline infusion (1 mL/h). All ketamine and saline bolus doses were delivered in a 1 mL/kg volume.

### Delayed ketamine experiment

Following the immediate ketamine experiment, a separate cohort of animals was tested with a delayed ketamine infusion 1 day after the fear conditioning. The ketamine dose (10 mg/kg over 2 h) was chosen based on the maximum effects on fear memory from the immediate ketamine experiment. Animals were randomly assigned into two groups (0 and 10 mg/kg, *n* = 8 per group). One day after the fear conditioning, the ketamine group received a bolus (2 mg/kg) and a 2-h ketamine infusion (10 mg/kg), whereas the saline group received a saline bolus and saline infusion (1 mL/h) (Fig. 1B). The ketamine and saline boluses were delivered in a 1 mL/kg volume.

### Ketamine injection experiment

In a separate study, animals were randomized into groups and were injected with a single ketamine (0 and 10 mg/kg, IP, *n* = 7 per group) immediately after fear conditioning (day 0). Fear memory retrieval, fear extinction, and fear recall were tested on days 2, 3, and 4, respectively (Fig. 1).

### Fear memory retrieval, extinction, and recall

Fear memory retrieval and extinction were tested in a separate chamber (Context B) constructed with Plexiglas and aluminum walls (Coulbourn Instruments, Lehigh Valley, PA) (Fig. 1). The chamber consisted of altered geometry (slanted Plexiglas ceiling) and spatial cues (red and black tape on walls), a plastic floor covered with a thin layer of shredded wood chip bedding, and a novel odorant (1% acetic acid). A dim light illuminated the chamber for observation and an infrared video camera on the ceiling facilitated video recording of animals. A wall-mounted speaker delivered the auditory stimuli. Context B was housed inside a larger sound-attenuating box (Coulbourn Instruments, Lehigh Valley, PA) with a background noise level of 55 dB.

Animals underwent fear retrieval and extinction testing as previously described^16^. After an acclimation period of 180 s, animals received 12 auditory tone presentations (5 kHz, 75 dB, 20 s; ITI 90–120 s) in Context B. Freezing was defined as no body movement, except as required for respiration, and was expressed as a percent freezing during the 20-s tone presentation. Freezing time was averaged and presented in blocks of two trials (six blocks in total). Contextual and cued fear recall were tested on day 4 (Fig. 1). Animals were placed in the fear conditioning chamber (Context A) and freezing to the context was measured for 180 s. At the conclusion of the 180-s period, a single auditory tone (5 kHz, 75 dB, 20 s) was played and freezing was measured for an additional 300 s. Two observers blind to the treatment condition scored the freezing time and the freezing time was converted to a percent freezing during the recording period.

### FDG-PET and CT imaging

In a separate study, animals were randomly assigned into two groups (0 and 10 mg/kg, *n* = 8 per group). Three FDG-PET and computed tomography (CT) scans were obtained for each animal at the following time points: (a) a baseline scan, (b) immediately after fear conditioning and 2-h ketamine infusion (Scan 1), and (c) immediately after fear memory testing (Scan 2) as shown in Fig. 1C. PET and CT images were acquired using an Inveon multimodality preclinical scanner (Siemens Medical Solutions, Erlangen, Germany) in the small animal PET and CT facility as described previously^34^. Animals were briefly anesthetized with isoflurane (4% induction and 1.5–2.5% maintenance) and injected IV with 1.7 mCi ± 0.153 mCi (62.9 MBq ± 5.7 MBq) ^18^F-FDG. After the injection of ^18^F-FDG, each animal was returned to a clean cage during the conscious uptake period (30 min) in a quiet room adjacent to the PET and CT scanner. Animals were undisturbed and exhibited minimal movement in the cages during the ^18^F-FDG uptake period. After the uptake, all animals were anesthetized with isoflurane to perform PET and CT scans.

Physiologic monitoring included measurements of temperature, respiration rate, heart rate, and oxygen saturation. PET data were acquired with a coincidence-timing window (Dt) of 3.4375 ns and energy window (De) of 350–650 keV in a list mode for 30 min. PET data were reconstructed as a single, high-resolution static frame using a 3D-ordered subsets expectation maximization/maximum a posteriori (3D-OSEM/MAP) algorithm (2 OSEM iterations, 18 MAP iterations, requested resolution: 0.8 mm) with scatter, attenuation, and decay corrections applied. PET image dimensions were 256 × 256 × 159 with a voxel size of 0.39 × 0.39 × 0.80 mm. Following the PET scan, a two-bed CT scan was acquired for anatomical localization, attenuation, and scatter correction (80 kvP, 500 µAs, 435 ms, 220° rotation). CT data were reconstructed in real time using a Feldkamp cone beam algorithm (0.5 mm Shepp filter) and corrected for beam hardening and Hounsfield Unit (HU). The CT image dimensions were 384 × 384 × 336 with a voxel size of 0.22 mm^3^ isotropic.

### FDG-PET data analysis

Image processing and analysis of the FDG-PET data were performed using VivoQuant software (ver 2.1, inviCRO LLC, Boston, MA) as described previously^34^. FDG-PET data were resampled to match CT voxel size (0.22 mm^3^ isotropic) and dimensions (384 × 384 × 336). The PET data were converted to units of activity (µCi) and registered to the CT image. Co-registered PET and CT images were uniformly cropped to a region surrounding the brain (170 × 170 × 240) and automatically registered to a 13-region rat brain atlas using an algorithm that combines a rigid transformation of the data and scaling of the atlas. The 13 regions included basal ganglia, thalamus, amygdala, cerebellum, cortex, hypothalamus, midbrain, corpus callosum, olfactory, hippocampus, septal area, white matter, and ventricles. The standard uptake value (SUV) of each region was normalized with the SUV of the entire atlas (whole-brain normalization) of the same animal.

### Statistics

All data are presented as mean ± standard error of the mean (SEM) and are normally distributed with similar variances across groups by D’Agostino & Pearson normality test. The sample size per group was determined based on a previous work^35^ and with a two-tailed significance of *α* = 0.05 and *β* = 80%. A two-way repeated-measures (RM) analysis of variance (ANOVA) with ketamine and time (RM) as independent variables was used to analyze horizontal activity, fear extinction, and BGluM data. A one-way ANOVA was used to analyze the area under the curves (AUCs) and fear memory recall in the immediate ketamine experiment. A two-tailed *t*-test was used for the AUC and fear memory recall in the delayed ketamine and IP ketamine experiments. Holm-Sidak’s post-hoc tests were used to compare group differences following the two-way RM ANOVA. Data that exceeded two standard deviations from the mean were treated as outliers and excluded from analysis. Data were analyzed using GraphPad Prism (GraphPad Software, version 7.0) and the accepted level of significance was *p* *<* 0.05.

## Results

### Immediate ketamine experiment

#### Locomotor activity

Horizontal locomotor activity was monitored during the infusion period immediately after the fear conditioning. There was a significant main effect of ketamine *F* (3, 36) = 11.54 (*p* *<* 0.001) on horizontal activity. Post-hoc tests showed that 10 mg/kg ketamine significantly reduced horizontal activity at all time points as compared with the saline group (Fig. 1D). The activity between 10 mg/kg dose and other doses (2 and 20 mg/kg) were significant between 100- and 120-min period (*p* < 0.05).

#### Fear memory retrieval, extinction, and recall

**Fig. 2:**
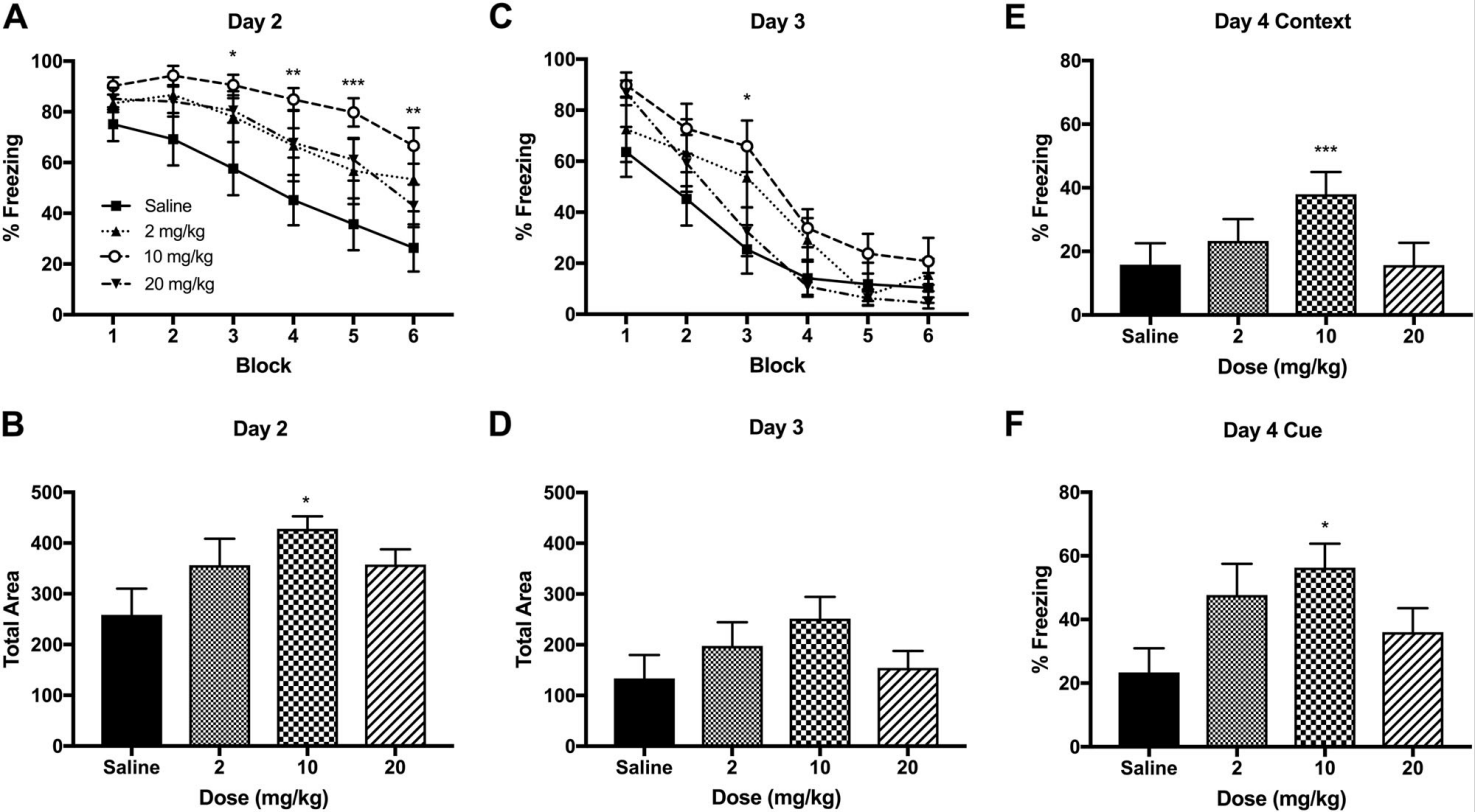
The effects of an immediate IV ketamine infusion on fear memory in rats. **A** The ketamine group (10 mg/kg) exhibited a significant delay in fear extinction when tested 2 days after ketamine infusion. Fear memory extinction was tested with 12 auditory tone trials (each block represents an average of two trials) in novel Context B (days 2 and 3). **B** The 10 mg/kg dose significantly increased total freezing based on the area under the curve (AUC) calculation. **C** The second fear extinction test on day 3 indicates a rapid extinction across all groups and there were no significant differences at the end of the fear extinction. **D** The total freezing behaviors on day 3 based on the AUC calculation were not different between the groups. **E** The 10 mg/kg ketamine increased contextual fear recall on day 4. **F** The 10 mg/kg ketamine also increased cued fear recall on day 4. Contextual fear recall is expressed as % freezing of the initial 180-s in Context A and cued fear recall is expressed as % freezing of 300-s after a single 20-s auditory tone presentation (day 4). Data are shown as mean ± SEM (*p *<* 0.05, **p *<* 0.01, ***p *<* 0.001 vs. saline controls)

The effects of an immediate ketamine infusion after fear conditioning on fear memory retrieval and extinction were tested on day 2. There were significant main effects of ketamine *F* (3, 36) = 4.364 (*p* *<* 0.01) and time *F* (5, 180) = 34.31 (*p* < 0.001) on freezing behavior. Post-hoc tests indicated that 10 mg/kg ketamine significantly increased freezing behavior from the block 3 to 6 as compared with the saline group (Fig. 2A). The AUC calculation was used to measure the total freezing and there were significant differences between the groups on day 2 *F* (3, 20) = 3.264 (*p* *<* 0.05). Post-hoc tests revealed that ketamine 10 mg/kg increased total freezing as compared with the saline group (*p* < 0.05) (Fig. 2B). The 10 mg/kg dose induced a greater effect than those of 2 and 20 mg/kg doses, indicating an inverted U shape dose-response function on fear memory. The same test was repeated on day 3 to capture the full fear extinction in the animals. A two-way ANOVA indicated a significant dose × time interaction *F* (15, 180) = 1.832 (*p* *<* 0.05) and a main effect of time *F* (5, 180) = 93.28 (*p* *<* 0.001) on freezing behavior. Post-hoc tests revealed that 10 mg/kg ketamine was significant from the saline group in the block 3 (Fig. 2C). However, the analysis of AUC indicated no significant group differences on freezing on day 3 *F* (3, 20) = 1.493 (*p* *=* 0.25; Fig. 2D).

Fear memory recall was tested in the fear conditioning chamber (Context A) on day 4. A one-way ANOVA indicated a significant main effect of ketamine *F* (3, 33) = 7.56 (*p* *<* 0.001) for the context re-exposure (Fig. 2E). A one-way ANOVA also indicated a significant main effect of ketamine *F* (3, 33) = 3.19 (*p* < 0.05) for the cue re-exposure (Fig. 2F). Post-hoc tests revealed that 10 mg/kg ketamine significantly increased freezing to the context (*p* < 0.001) and to the cue (*p* < 0.05) when compared with the saline group.

### Delayed ketamine experiment

#### Locomotor activity

Horizontal activity was monitored during the IV ketamine infusion (10 mg/kg), which was administered 1 day after the fear conditioning. There were significant main effects of ketamine *F* (1, 14) = 17.26 (*p* < 0.001) and time *F* (5, 70) = 5.14 (*p* < 0.001) on horizontal activity (Fig. 1E). Post-hoc tests revealed that ketamine 10 mg/kg significantly reduced horizontal activity between the 40- and 80-min period as compared with the saline group (*p* *<* 0.01).

#### Fear memory retrieval, extinction, and recall

**Fig. 3:**
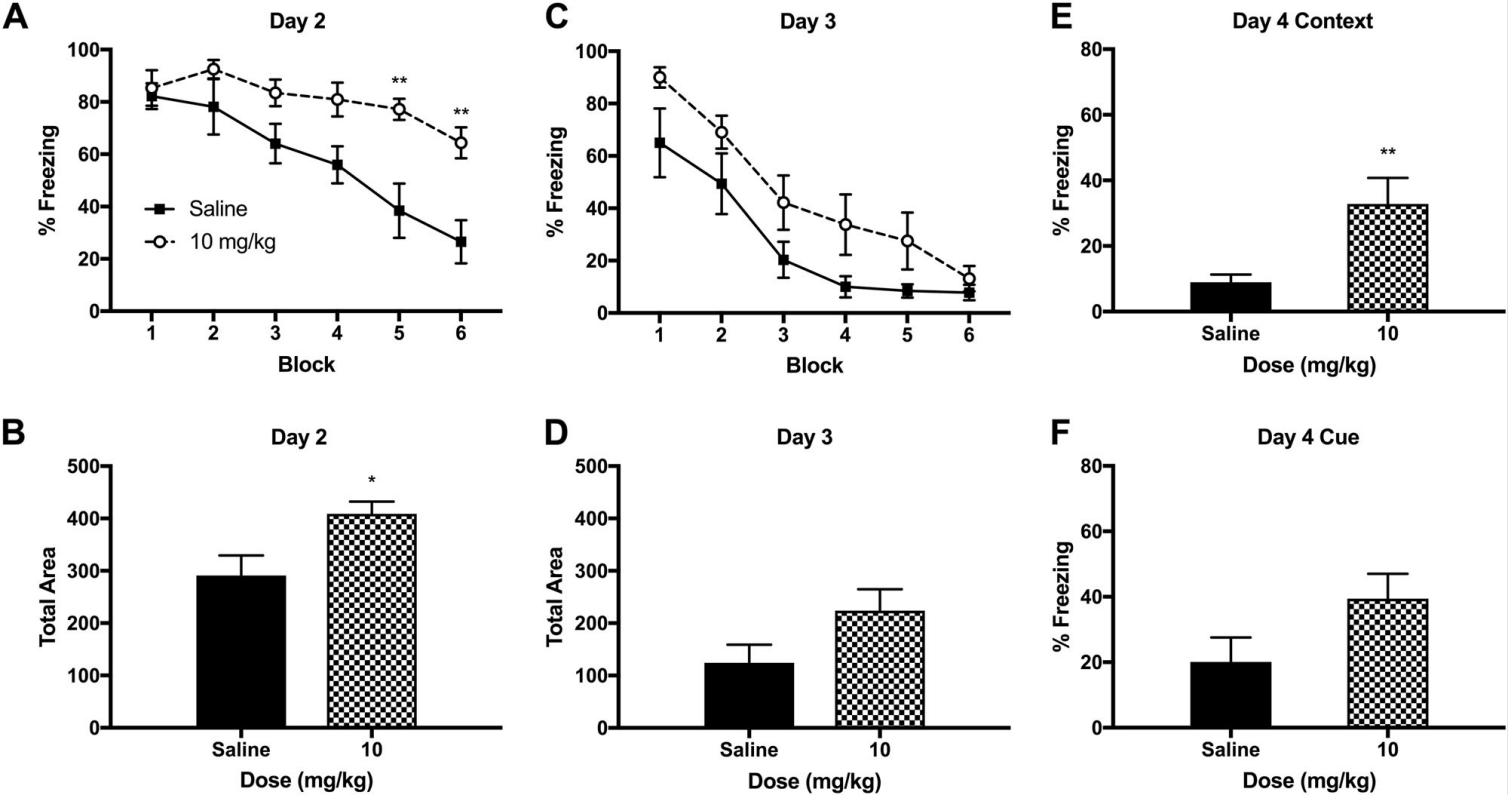
The effects of a delayed IV ketamine infusion on fear memory in rats. **A** The 10 mg/kg ketamine infusion significantly delayed cued fear extinction (day 2). Fear retrieval and extinction were tested over 12 auditory tone trials (each block represents an average of two trials) in novel Context B on days 2 and 3. **B** The 10 mg/kg dose significantly increased total freezing based on the AUC calculation. **C** The second fear extinction test on day 3 indicates a rapid extinction and there was no significant difference between the saline and the ketamine groups at the end of the testing. **D** There was no significant difference between the saline and ketamine groups on total freezing on day 3. **E** The ketamine group exhibited increased contextual fear recall as compared with the saline group on day 4. **F** However, cued fear recall was not significantly different between the groups. Contextual fear recall is expressed as % freezing of the initial 180-s in Context A and cued fear recall is expressed as % freezing of 300-s after a single 20-s auditory tone presentation. Data are shown as mean ± SEM (*p < 0.05, **p *<* 0.01 vs. saline controls)

The effects of a delayed ketamine infusion, administered 1 day after the fear conditioning, on fear memory retrieval and extinction were tested on days 2 and 3. There were significant main effects of ketamine *F* (1, 14) = 11.14 (*p* < 0.01), time *F* (5, 70) = 15.32 (*p* < 0.001), and a ketamine × time interaction *F* (5, 70) = 3.082 (*p* < 0.01) on freezing in day 2 (Fig. 3A). Post-hoc tests revealed increased freezing between the ketamine and saline groups in the blocks 5 and 6. Based on the AUC analysis, ketamine 10 mg/kg significantly increased freezing as compared with the saline group on day 2 *t* (10) = 2.61 (*p* *=* 0.03) (Fig. 3B). A two-way ANOVA indicated a significant main effect of time *F* (5, 70) = 41.12 (*p* *<* 0.001) on day 3 (Fig. 3C). However, the AUC analysis revealed no differences in total freezing between the ketamine and saline group on day 3 *t* (10) = 1.87 (*p* = 0.09) (Fig. 3D). Fear memory recall test on day 4 indicated increased freezing to the context *t* (14) = 2.912 (*p* < 0.05) but not to the cue *t* (14) = 1.821 (*p* = 0.09) in the ketamine group as shown in Figs. 3E, F, respectively.

### BGluM

**Fig. 4:**
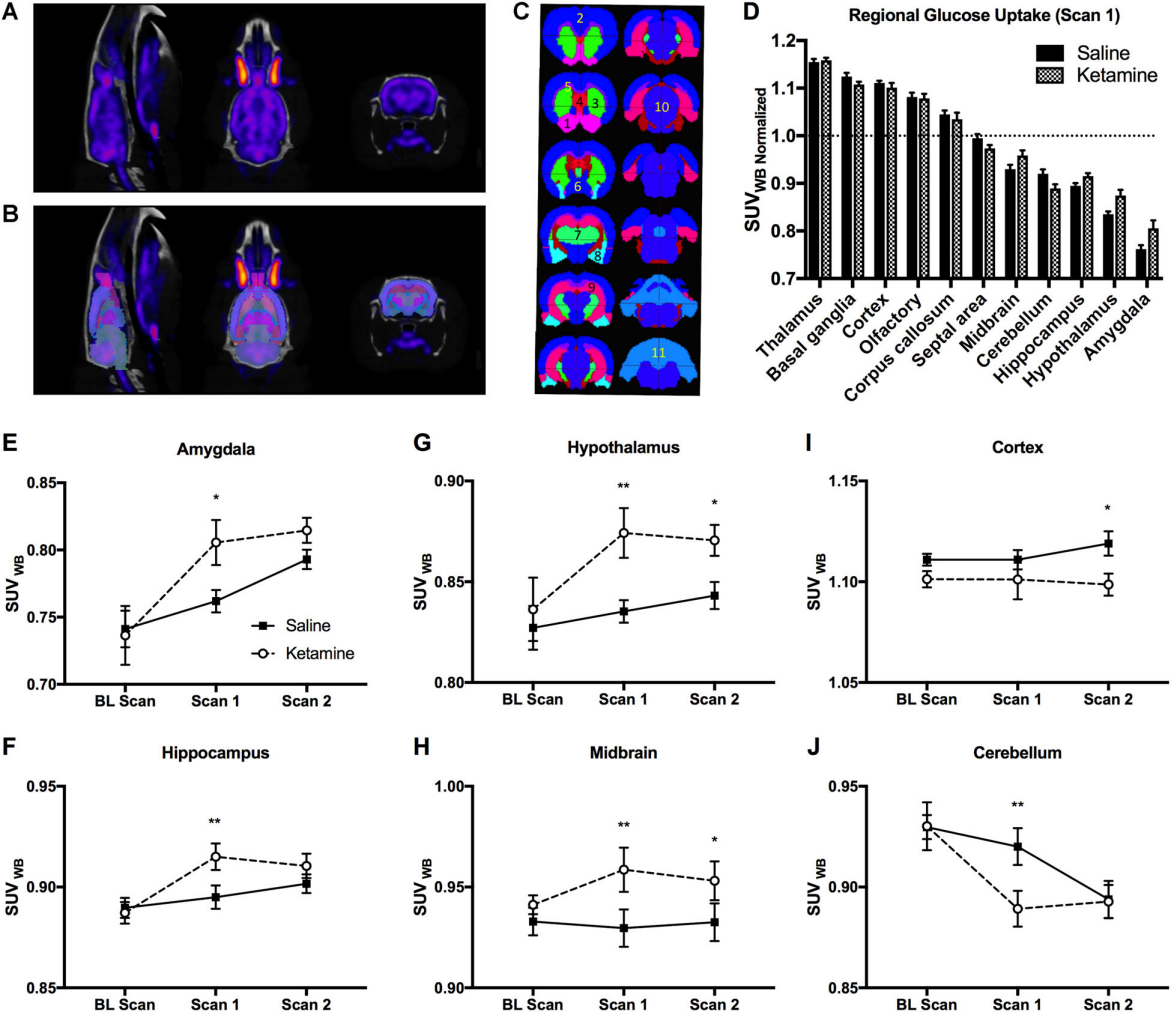
The effects of IV ketamine infusion on BGluM of rats. **A** Representative images of FDG-PET and CT of a rat brain (sagittal, planar, and coronal section, from left to right). **B** FDG-PET and CT images that are co-registered with the three-dimensional rat brain atlas. **C** Major brain regions quantified using the rat brain atlas. 1: Olfactory, 2: Cortex, 3: Basal Ganglia, 4: Septal Area, 5: Corpus Callosum, 6: Hypothalamus, 7: Thalamus, 8: Amygdala, 9: Hippocampus, 10: Midbrain, 11: Cerebellum. **D** IV Ketamine (10 mg/kg) infusion induced region-specific changes in BGluM as compared with the saline infusion. The IV Ketamine infusion (Scan 1) increased BGluM in the amygdala (**E**), hippocampus (**F**), hypothalamus (**G**), and midbrain (**H**), whereas decreasing it in the cerebellum (**J**). The fear memory testing (Scan 2) increased BGluM in the hypothalamus (**G**) and midbrain (**H**), whereas decreasing it in the cortex (**I**). SUV: standard uptake value. The SUV of each region was normalized with the SUV of the whole brain of the same animal. Data are shown as mean ± SEM (*p *<* 0.05, **p *<* 0.01 vs. saline controls)

^18^F-FDG-PET data were analyzed using the rat brain atlas method and representative images of FDG-PET and CT are shown in Fig. 4A. Figure 4B illustrates the same FDG-PET and CT images that were co-registered to the rat brain atlas. Figure 4C indicates 11 major brain regions that were quantified for BGluM in the rat brain. The effects of IV ketamine infusion (10 mg/kg) on BGluM in 11 regions are shown in Fig. 4D. A two-way ANOVA indicated a significant ketamine × brain region interaction *F* (10, 154) = 3.89 (*p* *<* 0.01) and a main effect of brain region *F* (10, 154) = 352.3 (*p* < 0.001) on BGluM. The BGluM levels were higher in certain regions such as in the thalamus, basal ganglia, cortex, olfactory, and corpus callosum as compared with the whole-brain BGluM levels.

The IV ketamine infusion (10 mg/kg) following fear conditioning increased BGluM in the amygdala (Fig. 4E), hippocampus (Fig. 4F), hypothalamus (Fig. 4G), and midbrain (Fig. 4H), while decreasing it in the cerebellum (Fig. 4J) (*p* < 0.05) (Scan 1). There were significant main effects of time in the amygdala *F* (2, 14) = 9.042 (*p* < 0.01), hippocampus *F* (2, 14) = 9.346 (*p* < 0.01), and cerebellum *F* (2, 14) = 15.59 (*p* < 0.01). There were significant main effects of ketamine on the BGluM in the midbrain *F* (1, 7) = 6.13 (*p* < 0.05), hypothalamus *F* (1, 7) = 7.664 (*p* < 0.05), and cortex *F* (1, 7) = 6.129 (*p* < 0.05). The fear memory testing (Scan 2) increased BGluM in the midbrain (Fig. 4H) and hypothalamus (Fig. 4G), while decreasing it in the cortex (Fig. 4I) as compared with the saline controls.

### Ketamine injection

**Fig. 5:**
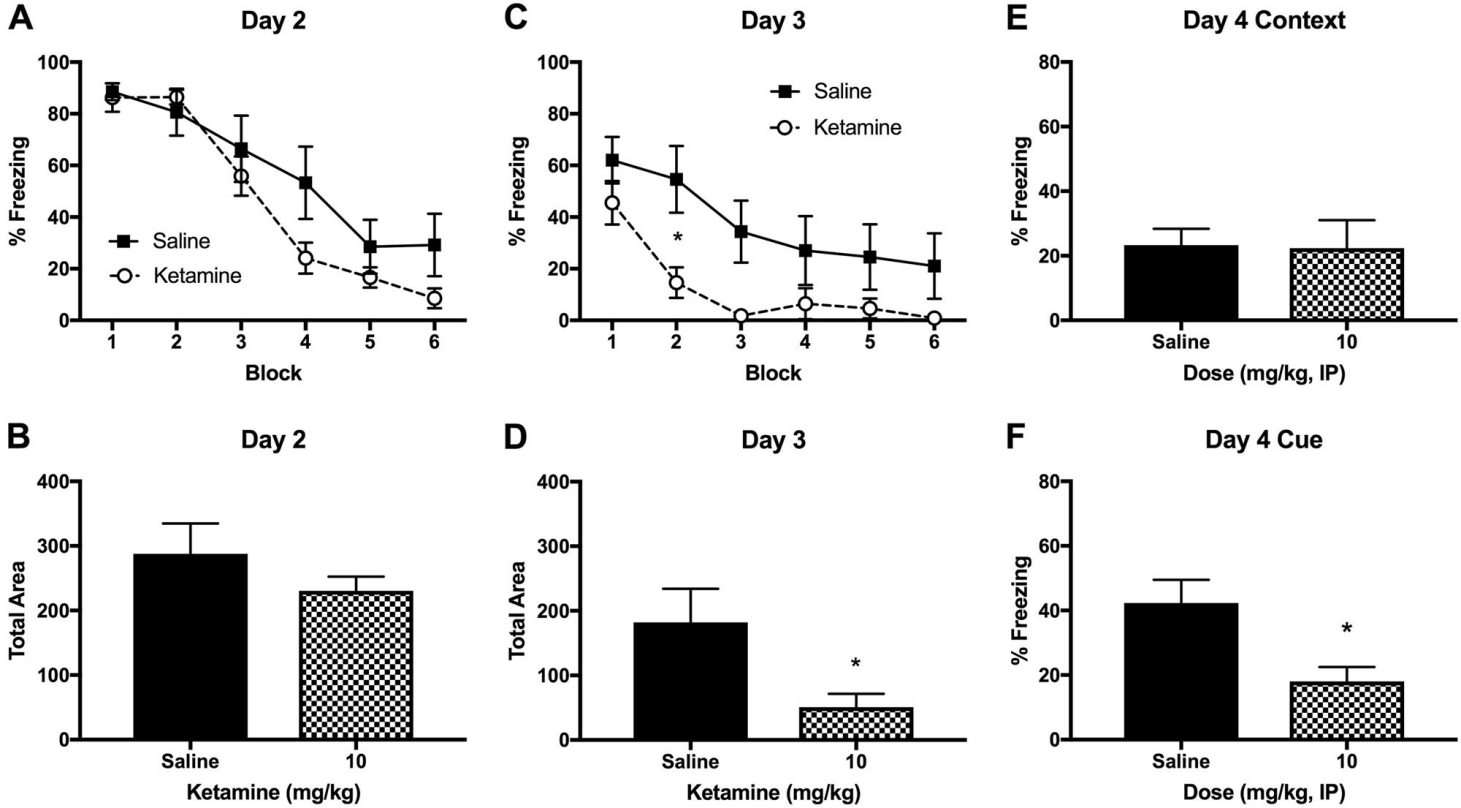
The effects of an IP ketamine injection (10 mg/kg) on fear memory in rats. **A** Fear memory retrieval and extinction was not significantly different between the ketamine and saline groups when the animals were tested first time (day 2). Each block represents an average of two tone trials in the Context B. **B** The area under the curve (AUC) was not different between the ketamine and saline groups (day 2). **C** The ketamine group showed a facilitated fear extinction during the second cue test on day 3. **D** The AUC was significantly lower in the ketamine group as compared to the saline group on day 3. **E** The fear recall to the context A (fear conditioning chamber) was not different between the ketamine and saline groups on day 4. **F** The fear recall to the auditory tone (CS) was significantly reduced in the ketamine group as compared to the saline group on day 4. Contextual fear recall is expressed as % freezing during the initial 180 s in Context A and cued fear recall is expressed as % freezing during the 300 s after the single 20-s auditory tone presentation (day 4). Data are shown as mean ± SEM (*p *<* 0.05 vs. saline controls)

A single ketamine injection (10 mg/kg, IP) after fear conditioning had no effects on fear memory retrieval on day 2 (Figs. 5A, B), but facilitated fear memory extinction on day 3 (Fig. 5C). A two-way RM ANOVA indicated a significant main effect of ketamine *F* (1, 12) = 5.096 (*p* < 0.05) and time *F* (5, 60) = 17.02 (*p* < 0.001) on day 3. Post-hoc tests revealed that the block 2 was significantly different between the ketamine and saline groups (*p* < 0.05). The AUC analysis revealed that total freezing was significantly lower in the ketamine group *t* (12) *=* 2.352, *p* < 0.05 (Fig. 5D). Fear recall to the context was not different between the groups (Fig. 5E) but fear recall to the cue was significantly reduced in the ketamine group as compared to saline group *t* (12) = 2.872, *p* < 0.05 (Fig. 5F).

## Discussion

Ketamine is administered to trauma victims to provide sedation and analgesia following injury. Although a seemingly ideal trauma anesthetic and analgesic due to its cardio-pulmonary stability and high-safety ceiling^1^, ketamine also induces adverse effects such as hallucination, delusion, and dissociation^3,36^ that may negatively impact the psychological health of trauma victims. Differences in methodology and outcomes between preclinical and clinical studies of ketamine make direct comparison of those investigations difficult. Although the IV route is preferred in most patients, rodent studies exclusively use ketamine IP injections that do not maintain a steady plasma ketamine levels over time. Therefore, we utilized a continuous IV ketamine infusion in freely moving rats to investigate the impacts on fear memory and in vivo BGluM. We found that IV ketamine infusion dose-dependently enhanced fear memory and the 10 mg/kg dose increased BGluM in brain regions that are critical for fear and stress responses. Interestingly, the same dose of ketamine IP injection produced an opposite effect on fear memory. To our knowledge, this is the first study reporting the effects of IV ketamine infusion on fear memory and in vivo brain energy utilization in rodents.

A ketamine infusion (10 mg/kg) suppressed spontaneous locomotor activity, indicating sedative effects of ketamine at this dose. This is consistent with our previous findings^27^ and others that also observed reduced locomotor activity following a low-dose ketamine injection^37–39^. In comparison, high doses of ketamine injection generally increase locomotor activity, indicating prominent dissociative stereotypy in animals^40,41^. Therefore, it is important to note that the IV ketamine doses (10 and 20 mg/kg over 2 h) tested in the current study is analgesic without severe dissociative stereotypy in rats.

The 10 mg/kg infusion administered immediately after fear conditioning enhanced fear memory retrieval and delayed fear extinction. Previous studies have reported inconsistent findings on the effects of ketamine on fear memory in rodents. For example, a ketamine IP injection to rodents either impaired^42,43^, had no effect^21^, or enhanced fear memory retrieval^4,44^. Other studies reported impaired fear extinction with NMDA receptor antagonists, including MK-801 and phencyclidine^45–47^. A partial NMDA receptor agonist, d-cycloserine, enhanced fear extinction^48^, which further supports the role of glutamatergic signaling in fear extinction. In order to investigate the timing of ketamine infusion after fear conditioning, we delayed the ketamine infusion by 1 day after the fear conditioning. A delayed ketamine infusion also enhanced fear memory and delayed fear extinction, similar to the effects of immediate ketamine infusion on fear memory. This suggests that the IV ketamine infusion (2 h) may have relatively long-lasting effects on fear memory consolidation. Recent studies also reported enhanced fear memory retrieval after sub-anesthetic doses of ketamine injection in rodents^19,49^. Therefore, previous studies and the current findings suggest that ketamine doses, routes and duration of administration may cause differential effects on fear memory consolidation and expression.

Both immediate and delayed ketamine infusion after fear conditioning enhanced fear memory recall to the context. However, only the immediate, not delayed, ketamine infusion following fear conditioning enhanced fear recall to the auditory cue (CS). This implies that an immediate ketamine infusion after fear learning may strengthen fear memory consolidation coinciding with the amygdala activation period. The delayed ketamine infusion may have produced a weaker effect on fear memory consolidation because the timing of ketamine administration was one day after the fear learning.

The IV ketamine (10 mg/kg) infusion altered in vivo BGluM in rats. The ketamine infusion decreased BGluM in the cerebellum of rats, which agrees with a clinical investigation that reported dissociative symptoms and decreased BGluM in the cerebellum of human subjects following IV ketamine infusion (1.2 mg/kg)^31^. Therefore, reduced BGluM in the cerebellum may indicate sedative and mild dissociative effects of ketamine infusion. A previous study using FDG-PET imaging found that 10 pairings of auditory tone and foot shock increased BGluM in the amygdala of rats^50^. In the current study, there was a trend of increased BGluM in the amygdala and hypothalamus of saline control animals (baseline scan vs. Scan 1). This may be due to a poor spatial resolution of FDG-PET imaging in small animals and this FDG-PET method may not be sensitive enough to detect mild fear conditioning effects. However, the IV ketamine infusion (10 mg/kg) produced robust effects on BGluM in several brain regions when compared with the saline controls (Scan 1). The hippocampus, amygdala, and hypothalamus showed increased BGluM, and enhanced activity of these regions may contribute to the enhanced fear memory observed in the ketamine group. A recent FDG-PET study reported that a post-fear conditioning ketamine injection (10 mg/kg, IP) increased fear behavior but did not alter BGluM in the hippocampus and amygdala of rats^20^. The authors measured BGluM 2 days after ketamine injection, whereas we measured BGluM immediately after the IV ketamine infusion in rats. Thus, it is possible that ketamine-induced BGluM changes may not last up to 2 days.

On the contrary to the IV ketamine infusion, an IP ketamine injection (10 mg/kg) following fear conditioning facilitated fear extinction (day 3) and reduced fear memory recall (day 4). Interestingly, the IP ketamine injection did not alter fear memory retrieval (day 2), indicating specific effects on fear memory extinction. Our findings are in line with a previous study that reported facilitated fear extinction following a ketamine injection (10 mg/kg, IP) in rats^51^, suggesting a facilitated extinction learning by IP ketamine injection. The current findings highlight the significance of the route (IV vs. IP) and duration (2 h vs. a bolus) of ketamine administration on fear memory processing. These results have clinical implications because patients may receive either a bolus (intranasal, intramuscular, or intravenous) and/or an IV infusion of ketamine depending on the clinical indication^52^.

The current study is not without limitations. First, we utilized a compressed timeline to assess fear memory retrieval, extinction, and recall in a 5-day period. Thus, our findings are more likely indicative of ketamine effects on ASD in patients, which resemble PTSD-like symptoms within the first month after the trauma. Therefore, we cannot translate the current findings to PTSD in humans. Assessing fear memory retrieval and extinction at extended time points after ketamine infusion (i.e., 1 or 2 week later) may be more relevant to PTSD. We did not administer midazolam, a benzodiazepine, commonly co-administered with ketamine in clinical settings to offset adverse dissociative effects. We anticipated that a co-administrated psychoactive agent with GABAergic properties may confound ketamine effects on fear memory. Lastly, we tested only male rats in the current study. Previous studies have shown sex-related differences in ketamine metabolism and antidepressant effects of ketamine in rodents^53–55^. Therefore, we cannot generalize our findings to female rats and a further study is warranted to investigate potential sex-related differences in ketamine effects on fear and stress related disorders.

In conclusion, sub-anesthetic doses of IV ketamine infusion dose-dependently enhanced fear memory retrieval and delayed fear extinction in rats. However, a ketamine IP injection following fear learning facilitated fear extinction, suggesting differential effects of ketamine depending on the route and duration of administration. The IV ketamine infusion enhanced BGluM in multiple brain regions that are critical for fear and stress responses. A further study is necessary to replicate the current findings and to investigate molecular mechanisms of IV ketamine infusion on fear memory and brain function.

## Disclaimer

The views expressed in this article are those of the authors and do not reflect official policy or position of the Uniformed Services University of the Health Sciences, Department of the Navy, the Department of Defense, or the United States Government.
